# Antibiotic resistance challenge: evaluating anthraquinones as rifampicin monooxygenase inhibitors through integrated bioinformatics analysis

**DOI:** 10.1186/s44342-024-00015-2

**Published:** 2024-09-04

**Authors:** Mohammad Reza Arabestani, Masoumeh Saadat, Amir Taherkhani

**Affiliations:** 1 Department of Microbiology, School of Medicine, Hamadan University of Medical Sciences, Hamadan, Iran; 2 Research Center for Molecular Medicine, Hamadan University of Medical Sciences, Hamadan, Iran

**Keywords:** Anthraquinone, Docking, Drug, Hypericin, Inhibitor, Rifampicin monooxygenase

## Abstract

**Objective:**

Antibiotic resistance poses a pressing and crucial global public health challenge, leading to significant clinical and health-related consequences. Substantial evidence highlights the pivotal involvement of rifampicin monooxygenase (RIFMO) in the context of antibiotic resistance. Hence, inhibiting RIFMO could offer potential in the treatment of various infections. Anthraquinones, a group of organic compounds, have shown promise in addressing *tuberculosis*. This study employed integrated bioinformatics approaches to evaluate the potential inhibitory effects of a selection of anthraquinones on RIFMO. The findings were subsequently compared with those of rifampicin (RIF), serving as a positive control inhibitor.

**Methods:**

The AutoDock 4.0 tool assessed the binding free energy between 21 anthraquinones and the RIFMO catalytic cleft. The ligands were ranked based on the most favorable scores derived from Δ*G*_binding_. The docking analyses for the highest-ranked anthraquinone and RIF underwent a cross-validation process. This validation procedure utilized the SwissDock server and the Schrödinger Maestro docking software. Molecular dynamics simulations were conducted to scrutinize the stability of the backbone atoms in free RIFMO, RIFMO-RIF, and RIFMO complexed with the top-ranked anthraquinone throughout a 100-ns computer simulation. The Discovery Studio Visualizer tool visualized interactions between RIFMO residues and ligands. An evaluation of the pharmacokinetics and toxicity profiles of the tested compounds was also conducted.

**Results:**

Five anthraquinones were indicated with Δ*G*_binding_ scores less than − 10 kcal/mol. Hypericin emerged as the most potent RIFMO inhibitor, boasting a Δ*G*_binding_ score and inhibition constant value of − 12.11 kcal/mol and 798.99 pM, respectively. The agreement across AutoDock 4.0, SwissDock, and Schrödinger Maestro results highlighted hypericin’s notable binding affinity to the RIFMO catalytic cleft. The RIFMO-hypericin complex achieved stability after a 70-ns computer simulation, exhibiting a root-mean-square deviation of 0.55 nm. Oral bioavailability analysis revealed that all anthraquinones except hypericin, sennidin A, and sennidin B may be suitable for oral administration. Furthermore, the carcinogenicity prediction analysis indicated a favorable safety profile for all examined anthraquinones.

**Conclusion:**

Inhibiting RIFMO, particularly with anthraquinones such as hypericin, holds promise as a potential therapeutic strategy for infectious diseases.

**Supplementary Information:**

The online version contains supplementary material available at 10.1186/s44342-024-00015-2.

## Introduction

Antibiotic resistance is an urgent and critical global public health challenge, exerting profound clinical- and health-related repercussions. The proliferation and dissemination of bacteria strains exhibiting antibiotic resistance present formidable obstacles to effectively managing diseases and delivering optimal patient care [1]. The imprudent and excessive employment of antibiotics in human healthcare and agricultural practices amplifies the genesis and diffusion of antibiotic resistance [1, 2]. Notably, the ascent of multidrug-resistant bacterial strains has emerged as a grave menace, precipitating elevated morbidity, mortality, and healthcare expenditures [3]. Furthermore, the potential transmission of antibiotic resistance from environmental sources to human populations is a subject of significant apprehension. The profligate use of antibiotics and antibiotic residues within the environment can contribute to the selection and dissemination of antibiotic-resistant bacteria and the corresponding resistance genes [2]. In response to this escalating crisis, combining therapy integrating approved antibiotics with inhibitors targeting innate resistance mechanisms has been advanced as a strategic initiative in the ongoing battle against antibiotic resistance [4].

Rifampicin (RIF), known as rifampin, initially gained approval for treating *tuberculosis*. Its widespread application in combination therapy for non-mycobacterial infections is due to its low toxicity, broad-spectrum effectiveness, and favorable bioavailability [5]. In the context of *tuberculosis* treatment, extensive research has explored its mechanism of action and pharmacokinetics. RIF functions by inhibiting bacterial RNA polymerase with a specific target on the enzyme’s beta subunit. This inhibition hampers bacterial mRNA transcription, disrupting protein synthesis and eventually leading to bacterial cell demise. Rifampicin demonstrates high efficacy against mycobacteria, notably *Mycobacterium tuberculosis*, the *tuberculosis*-causing agent. Concerning pharmacokinetics, rifampicin exhibits notable variability among individuals. However, recent studies have raised concerns about decreased cure rates, potentially linked to an increased emergence of resistance [6].

Accumulating evidence indicates the pivotal role of rifampicin monooxygenase (RIFMO) in antibiotic resistance. RIFMO catalyzes a chemical transformation of the RIF, converting it into a less effective form termed 2′-N-hydroxy-4-oxo-rifampicin [7]. Researchers have delved into understanding RIFMO’s mechanism. In a study by Liu et al. [8], the structural configuration of RIFMO, bound to the hydroxylated RIF product, was elucidated. This revealed a specific modification process wherein RIFMO introduces a hydroxyl group at the C2 atom of RIF, followed by the cleavage of a critical chemical bond known as the ansa linkage. This process effectively deactivates the antibiotic by impeding its interaction with RNA polymerase, the target of RIF.

Anthraquinones are naturally occurring compounds structurally similar to anthracene, granting them a broad spectrum of biological activities. These activities range from anticancer to anti-inflammatory effects. The mechanisms underlying these activities are complex and multifaceted, often involving interactions with various cellular pathways. For example, some anthraquinones induce apoptosis via caspase activation and modulate inflammatory cytokine pathways [9, 10]. A pivotal study highlighted the anticancer properties of anthraquinones like emodin and physcion, demonstrating efficacy against various cancer cell lines, including those of the breast, lung, and liver [11, 12]. The safety profile of anthraquinones is crucial. Diacerein, an anthraquinone derivative used in osteoarthritis treatment, exemplifies this challenge. While it exhibits modest pain reduction efficacy, it has been associated with gastrointestinal adverse events [12, 13]. Anthraquinones have shown promise in addressing *tuberculosis*, and in a study led by Sturdy et al. [14], eucapsitrione, an anthraquinone derivative from cyanobacterium *Eucapsis* sp., demonstrated anti-*M*. *tuberculosis* activity with MIC values of 3.1 and 6.4 micro M in relevant assays indicates its potential as an anti-*tuberculosis* agent [14]. A review article by Pollo et al. [15] examined 52 natural and synthetic compounds for *M*. *tuberculosis* activity, with certain anthraquinones, such as damnacanthal and isobavachalcone, showing antimycobacterial properties, affirming the potential of anthraquinones as a novel class of antimicrobial drugs for *tuberculosis* treatment.

The recent legislation passed in late December 2022 and endorsed by President Joe Biden has abolished the mandatory requirement for animal testing in the evaluation process for new pharmaceuticals seeking approval from the US Food and Drug Administration (FDA) [16]. This significant policy shift represents a transformative development, addressing a long-standing aspiration of animal welfare advocacy groups. It signifies a substantial departure from the historical reliance on animal experimentation that has characterized drug development for over eight decades. This momentous change underscores the need for scientists to embrace alternative methodologies, such as computer modeling, organ chips, and other innovative nonanimal approaches that have emerged and matured over the past 10 to 15 years. This reflects the growing confidence in the reliability and viability of computational results in drug design and discovery.

The computational approach to drug discovery (CADD) has revolutionized the field. This approach enables rapid screening and synthesis of vast compound libraries, addressing the historical challenges of low efficacy and high failure rates in drug development [17–21]. CADD encompasses a variety of techniques, including molecular docking, binding free energy analysis, molecular dynamics (MD) simulation, and pharmacokinetics analysis [22–24]. Molecular docking is a computer-aided screening method investigating ligand binding within protein target active sites [22]. While docking algorithms utilize scoring functions for swift evaluation of binding affinities, these functions often have limited correlation with experimental results [25]. More sophisticated methods for binding affinity evaluation have been developed, such as free energy perturbation (FEP), thermodynamic integration (TI), and molecular mechanics energies combined with Poisson-Boltzmann (MM/PBSA) or generalized born and surface area continuum solvation (MM/GBSA) [26–28]. MM/GBSA and MM/PBSA offer a valuable balance between speed and reliability compared to scoring functions and FEP/TI [25, 28]. MD simulation examines the stability and rigidity of protein–ligand interactions over time [23]. Integrating docking, free energy calculation, and MD simulation presents a robust approach for investigating protein–ligand interactions in drug discovery. Pharmacokinetics analysis plays a crucial role in preclinical studies, evaluating the effects of novel drug compounds on living organisms upon administration [29].

The rationale behind this study was firmly rooted in addressing the escalating global threat of antibiotic resistance. Extensive evidence underscores the pivotal role played by RIFMO in the emergence and dissemination of antibiotic resistance, rendering it a promising target for therapeutic intervention. Previous studies have showcased certain natural compounds’ antimicrobial and anti-*tuberculos*is properties, notably anthraquinones [14, 15]. Therefore, we postulated that a systematic exploration of anthraquinone compounds could unveil potent RIFMO inhibitors capable of combating antibiotic resistance. This research used integrated computational methodologies [23, 30, 31], including molecular docking and molecular dynamics (MD) simulations, to methodically evaluate the inhibitory impacts of a curated set of 21 anthraquinones [32, 33] on the RIFMO enzyme. The ultimate objective was to identify novel lead compounds that could be foundational in developing more efficacious antimicrobial therapies.

## Materials and methods

### Structural preparation of molecules

The structural data for the RIFMO protein (PDB ID: 5KOX) was sourced from the RCSB database, with an X-ray resolution of 1.8 Å [7, 34]. The PDB file included a single polypeptide chain, chain A, encompassing 473 residues. Energy minimizing was executed using Swiss-PdbViewer version 4.1.0, available at https://spdbv.unil.ch, to attain the enzyme’s most energetically stable conformation. To identify the critical residues within the active site of RIFMO, a comprehensive analysis of the interacting residues between the enzyme and the RIFMO inhibitor, rifampicin (PDB ID: RFP), was conducted. Additionally, the primary research study conducted by Liu et al. [7], which pertains to this protein, was thoroughly reviewed. As a result of this investigation, a set of pivotal residues were elucidated, which include Arg43, His46, Phe69, Val71, Phe74, Val93, Leu176, Arg201, Ile215, Phe256, Pro283, Thr284, Gly285, and Leu341. These residues’ geometric positioning within the receptor led to the establishment of specific grid box parameters: X-center, 8.97; Y-center, 28.564; Z-center, − 25.85; X-dimension, 82; Y-dimension, 60; and Z-dimension, 60.

In pursuing potential RIFMO inhibitors, a careful selection of 21 anthraquinones was curated [35]. The objective was to evaluate the binding affinities of these anthraquinones in comparison to RIF (PubChem ID, 135,398,735), which was employed as a reference drug sourced from the DrugBank repository ([https://go.drugbank.com/]), with a DrugBank ID of DB01045 [36]. RIF was included in the study as a positive control due to its established role as a potent RIFMO inhibitor. The comparison aimed to provide a benchmark for the binding free energy of the studied anthraquinones relative to a known inhibitor. Building upon our earlier investigative work [32, 33], a critical step involved executing an energy minimization procedure on the anthraquinones. In preparation for the subsequent analysis, PDBQT files were meticulously generated. This process entailed adapting the enzyme’s structural representation to incorporate Kollman charges and polar hydrogen bonds while applying rotational motion and local charge adjustments to the ligands. MGL tools (16) were effectively leveraged to facilitate these operations.

### Molecular docking analysis

The docking analyses were conducted using a Windows-based personal computer featuring an Intel Core i7 processor, 32 GB of installed RAM, and a 64-bit system architecture [37]. The computation of Δ*G*_binding_ values, measured in kcal/mol, for anthraquinones, a positive control compound, and their interaction with the RIFMO binding site was performed employing the semiflexible docking technique available in AutoDock version 4.0 [38]. A comprehensive set of 50 conformations was systematically generated for each ligand using the Lamarckian genetic algorithm. This approach thoroughly assessed the binding affinities among the herbal isolates, standard drug, and RIFMO active site. The docking results were organized into clusters using a root-mean-square (RMS) tolerance of 2.0 Å [39]. Subsequently, the most negative Δ*G*_binding_ value within the most prominent cluster was singled out for further evaluation. The BIOVIA Discovery Studio Visualizer tool generated molecular visualizations and analyzed interactions.

### Cross-validation study

The docking analyses underwent a cross-validation process for the top-ranked anthraquinone and RIF. This validation procedure employed the SwissDock server, accessible at http://www.swissdock.ch/ [40, 41], and the Schrödinger Maestro docking software, version 10.2 [42, 43]. SwissDock is a web-based service developed by researchers from the Molecular Modeling Group of the Swiss Institute of Bioinformatics in Lausanne, Switzerland, including Aurélien Grosdidier, Vincent Zoete, and Olivier Michielin. It allows users to predict potential molecular interactions between a target protein and a small molecule. SwissDock is built upon the docking software EADock DSS, following a specific workflow:

1. Generating potential binding modes within a defined region (local docking) or across all target cavities (blind docking).

2. Estimating the CHARMM energy of these binding modes on a grid.

3. Evaluation of the binding modes with the most favorable energies using the FACTS scoring function, followed by clustering.

4. Visualization and downloadable access to the most promising clusters of binding modes for further analysis.

Moreover, the Schrödinger Maestro tool enabled the Glide docking system to evaluate the binding affinity of the primary anthraquinone and RIF to the RIFMO active site. Within this framework, docking scores (G scores) were computed for the ligands [44]. The relative binding free energies between the compounds were also determined using the Prime MM-GBSA method.

### Molecular dynamics simulations

The MD investigations were carried out for three distinct systems: (1) RIFMO in isolation, (2) RIFMO complexed with its most potent inhibitor as determined from the molecular docking results, and (3) RIF, which served as a reference drug in the study. Further, the MD analyses were conducted through 100-ns (ns) computer simulations using the Discovery Studio Client tool, version 16.1.0.15350. To ensure the computational robustness of these simulations, a high-performance computer configuration was employed, featuring a 64-bit system, 64-GB DDR5 of installed RAM, and an Intel 24-Core i9-13900KF Processor. The advanced parameters for the MD simulations, encompassing parameters such as the cell shape, target temperature, solvation model, solvent, force field, and minimum distance from the boundary, adhered to the protocols established in our prior reports [45, 46]. During the MD simulations, several parameters were investigated, such as the root-mean-square deviation (RMSD) and the root-mean-square fluctuation (RMSF) of the RIFMO backbone atoms. The RMSF quantifies the average deviation of a particle, such as a protein residue, from its reference position over time. Additionally, the total energy and radius of gyration (ROG) of RIFMO were computed to enhance the reliability of the results.

### Bioavailability, pharmacokinetics, and toxicity of the compounds

The drug likeness of the anthraquinones and RIF was assessed based on Lipinski’s Rule of Five (Ro5), a widely recognized framework for evaluating the physiochemical properties of potential oral drug candidates [47]. This criterion, rooted in molecular characteristics, offers a comprehensive guideline for assessing drug candidacy. Utilizing the PubChem database, the chemo-physical properties of the ligands were meticulously examined. According to RO5, oral drug candidates should ideally not exceed one violation among four criteria: molecular mass ≤ 500 g/mol, logarithm of the partition coefficient between octanol and water (LogP) ≤ 5, number of hydrogen bond acceptors ≤ 10, and number of hydrogen bond donors ≤ 5.

Furthermore, the pharmacokinetic properties of the tested compounds were predicted through the SwissADME online platform (http://www.swissadme.ch/) [48]. This platform, encompassing absorption, distribution, metabolism, and excretion (ADME) modeling, offered valuable insights into crucial pharmacokinetic attributes such as gastrointestinal absorption potential, blood–brain barrier permeability, potential interactions with cytochrome P450 enzymes, and susceptibility as a P-glycoprotein substrate. Additionally, to gauge ligand carcinogenic potential as an indicator of compound toxicity, an assessment was conducted using the toxCSM web server (accessible at http://biosig.lab.uq.edu.au/toxcsm) [49].

## Results

### Potential RIFMO inhibitors

**Fig. 1:**
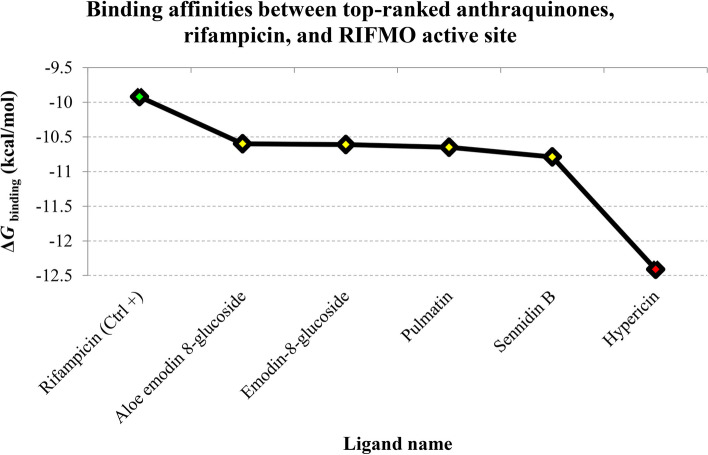
The Δ*G*_binding_ values represent the Gibbs free energy of binding, measured in units of kcal/mol, between the RIFMO catalytic cleft, the enzyme’s control inhibitor, and the top-ranked anthraquinones. The ligand names are displayed on the *X*-axis, while the corresponding Gibbs free binding energy is depicted on the *Y*-axis. A green diamond denotes the reference drug, and a red spot with a Ki value in the picomolar scale depicts the most potent inhibitor of RIFMO. Yellow spots indicate the other anthraquinones with Ki values in the nanomolar scale. RIFMO, rifampicin monooxygenase; *K*i, inhibition constant

According to the results, hypericin could restrict the RIFMO activity in a picomolar range, with the *K*i value and Δ*G*_binding_ score of 798.99 pM and − 12.41 kcal/mol, respectively. Thus, this anthraquinone was the most potent RIFMO inhibitor in this study. Besides, four other compounds, consisting of sennidin B, pulmatin (chrysophanol-8–*0*-glucoside), emodin 8-glucoside, and aloe-emodin 8-glucoside, demonstrated Δ*G*_binding_ score less than − 10 kcal/mol. As a result, these five herbal isolates were deemed the most robust inhibitors of RIFMO and were chosen for further interaction mode analysis. The Δ*G*_binding_ score of − 9.92 kcal/mol between RIF and RIFMO was identified. Consequently, the top-ranked anthraquinones in this study exhibited greater binding affinity to the RIFMO active site than the reference antibiotic. Supplementary File 1 provides details of docking result files for top-ranked anthraquinones and the reference drug. Table 1 provides an overview of the affinities between the tested compounds and RIFMO, displaying the calculated energies, including Δ*G*_binding_ and *K*i values between RIFMO and components. Figure 1 compares the binding affinities between the top-ranked RIFMO inhibitors in this study, the reference antibiotic, and the enzyme’s active site.

**Table 1:** Details of energies, including Δ*G*_binding_ and *K*i values, were determined for the interactions between the RIFMO, tested anthraquinones, and RIF, which served as the control antibiotic

PubChem ID	Ligand name	Final intermolecular energy (kcal/mol)	Final total energy (kcal/mol)	Torsional free energy (kcal/mol)	Unbound system’s energy (kcal/mol)	Estimated free energy of binding (kcal/mol)	*K*i
3663	Hypericin	− 10.46	− 4.59	2.39	− 0.25	− 12.41	798.99 pM
10459879	Sennidin B	− 12.53	− 4.59	3.88	− 2.45	− 10.79	12.31 nM
442731	Pulmatin (chrysophanol-8–*0*-glucoside)	− 8.93	− 6.21	2.98	− 1.5	− 10.65	15.54 nM
99649	Emodin 8-glucoside	− 9.38	− 5.96	3.28	− 1.44	− 10.61	16.69 nM
126456371	Aloe-emodin 8-glucoside	− 9.52	− 6.05	3.58	− 1.39	− 10.6	16.95 nM
10168	Rhein	− 9.05	− 2.33	2.09	− 0.07	− 9.22	174.64 nM
10639	Physcion	− 9.2	− 1.45	1.49	− 0.29	− 8.86	320.15 nM
92826	Sennidin A	− 12.61	− 5	3.88	− 4.9	− 8.84	332.62 nM
361510	Emodic acid	− 8.93	− 2.35	2.39	− 0.06	− 8.84	333.99 nM
6293	Alizarin	− 7.84	− 2.13	1.19	− 0.28	− 8.5	586.72 nM
10207	Aloe-emodin	− 8.48	− 2.1	1.79	− 0.33	− 8.46	629.92 nM
10208	Chrysophanol	− 7.87	− 2.15	1.19	− 0.37	− 8.46	633.85 nM
3220	Emodin	− 7.44	− 2.62	1.49	− 0.2	− 8.36	741.17 nM
6683	Purpurin	− 7.44	− 2.59	1.49	− 0.29	− 8.24	907.49 nM
101286218	Rhodoptilometrin	− 8.74	− 2.32	2.39	− 0.48	− 8.19	987.74 nM
2950	Danthron	− 7.84	− 1.97	1.19	− 0.5	− 8.12	1.11 µM
3083575	Obtusifolin	− 7.87	− 2.14	1.49	− 0.55	− 7.97	1.44 µM
442753	Knipholone	− 9.37	− 5.31	2.98	− 4.23	− 7.46	3.41 µM
124062	Rubiadin	− 8.08	− 1.28	1.19	− 1.28	− 6.89	8.96 µM
160712	Nordamnacanthal	− 8.4	− 2.12	1.79	− 2.15	− 6.58	15.00 µM
2948	Damnacanthal	− 7.86	− 1.6	1.79	− 1.25	− 6.42	19.72 µM
135398735	Rifampicin (Ctrl +)	− 12.47	− 0.16	1.79	− 0.92	− 9.92	53.32 nM

*RIFMO* rifampicin monooxygenase, *RIF* rifampicin, *K*i inhibition constant

### Cross-validations

The SwissDock server computed Δ*G*_binding_ values for hypericin, RIF, and the RIFMO catalytic cleft. Notably, hypericin exhibited a more negative binding free energy to the RIFMO active site than the reference drug, with Δ*G*_binding_ scores of − 8.62 and − 7.76 kcal/mol, respectively. Furthermore, Schrödinger Maestro software analysis supported this observation, indicating hypericin’s superior binding affinity to the RIFMO active site over the antibiotic, as indicated by G scores (Table 2). Consistency among results from AutoDock 4.0, SwissDock, and Schrödinger Maestro underscores hypericin’s significant binding affinity to the RIFMO catalytic cleft. Furthermore, the Prime MM-GBSA analysis revealed each compound’s relative binding-free energy (Δ*G*_binding_), with the results presented in Table 3.

**Table 2:** Docking scores (G scores) and Glide emodel scores from Schrödinger Maestro (in kcal/mol) were assessed for hypericin and RIF regarding their interaction with the RIFMO active site

Compound name	G score (dock score)	Glide emodel
Hypericin	− 7.883	− 59.42
RIF	− 7.579	− 60.32

*RIFMO* rifampicin monooxigenase, *RIF* rifampicin

**Table 3:** Relative binding free energies (kcal/mol) were determined by applying the prime MM-GBSA approach

Compound name	MMGBSA-dG-binding energy	MMGBSA-dG Bind in Coulomb	MMGBSA-dG Bind covalent	MMGBSA-dG Bind H-bond	MMGBSA-dG Bind (NS)	MMGBSA-dG Bind (NS) Coulomb
Hypericin	− 42.91	− 16.28	− 1.91	− 0.16	− 49.07	− 16.71
RIF	− 46.95	− 19.19	15.71	− 0.78	− 65.64	− 19.83

The MM-GBSA ΔG bind is calculated as the difference between the complex, receptor, and ligand energies. The MM-GBSA ΔG bind (NS) is derived from the complex-receptor (from optimized complex) and ligand (from optimized complex) energies, subtracting receptor strain and ligand strain. In the table, NS denotes the binding energy without accounting for the conformational changes of the receptor and ligand required for complex formation

### RIFMO stability

**Fig. 2:**
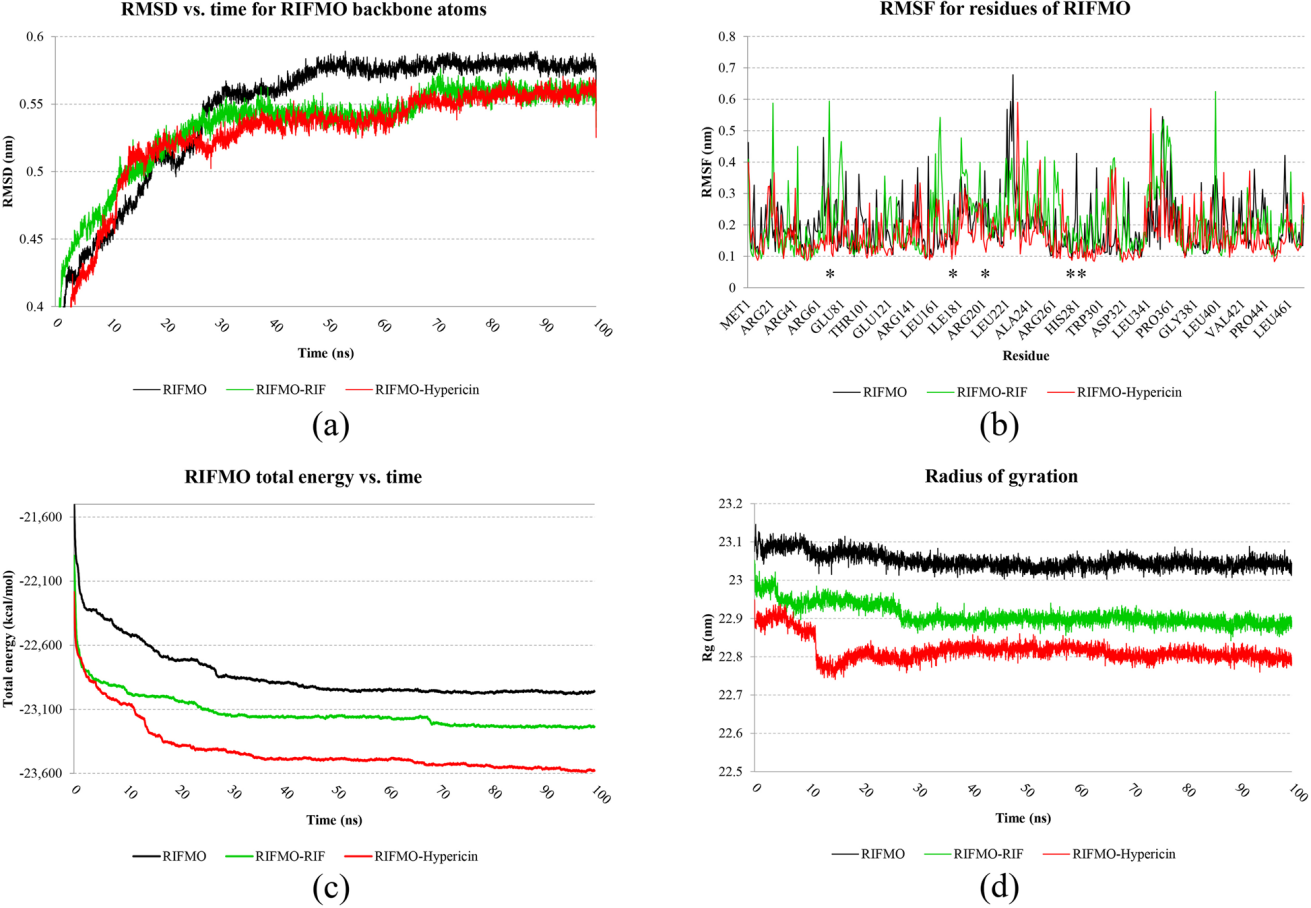
The impact of free RIFMO backbone atoms during a 100-ns MD simulation was studied with a focus on (**a**) RMSD, **b** RMSF, **c** total energy, and (**d**) ROG plots to examine the influence of hypericin and RIF. The asterisks in the RMSF plots denote the active site of the receptor. RIFMO, rifampicin monooxygenase; RIF, rifampicin; RMSD, root-mean-square deviations; RMSF, root-mean-square fluctuation; MD, molecular dynamics; ROG, radius of gyration

**Fig. 3:**
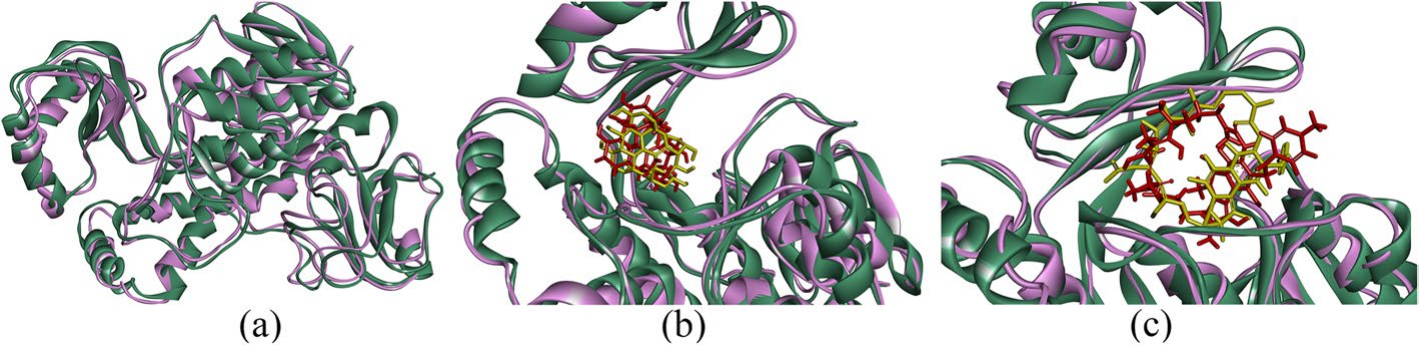
Following a 100-ns MD simulation, the superimposition of structures was performed for (**a**) free RIFMO, **b** RIFMO with hypericin, and (**c**) RIFMO with RIF. The protein chains were depicted in green and violet to indicate their states before and after the MD analyses. Additionally, the ligands were portrayed in yellow and red to represent their conditions before and after the MD simulations. RIFMO, rifampicin monooxygenase; RIF, rifampicin; MD, molecular dynamics

Following the root-mean-square deviation RMSD plot, discernible improvements in stability were observed in the receptor upon binding with hypericin and RIF, surpassing its unbound state. The data presented in Fig. 2a indicates comparable stability between RIFMO-hypericin and RIFMO-RIF. Consequently, the protein’s backbone atoms attain a state of equilibrium around the 70-ns mark, exhibiting an RMSD of approximately 55 nm when influenced by hypericin and RIF. The RMSF plots reveal diminished fluctuations in the active site of RIFMO when impeded by hypericin in contrast to RIFMO-RIF and its unbound state (as illustrated in Fig. 2b). As a result, it is inferred that the most potent inhibitor of RIFMO in this study conferred superior stabilization to the receptor compared to RIF. Based on the MD simulation findings depicted in Fig. 2c, it is evident that the total energy of the RIFMO complexed with hypericin was lower than that of free RIFMO and RIFMO-RIF. Concurrently, the ROG value for the RIFMO-hypericin complex, as illustrated in Fig. 2d, remained consistently lower than that of RIFMO-RIF and free RIFMO throughout the 100-ns MD simulation. Notably, the ROG value for RIFMO-hypericin was stable after undergoing a 20-ns simulation. Figure 3 visually displays the superimposed structures of free RIFMO, RIFMO complexed with hypericin, and RIFMO-RIF in pre- and post-computer simulations, as observed through the DSV tool.

### Interaction modes

**Fig. 4:**
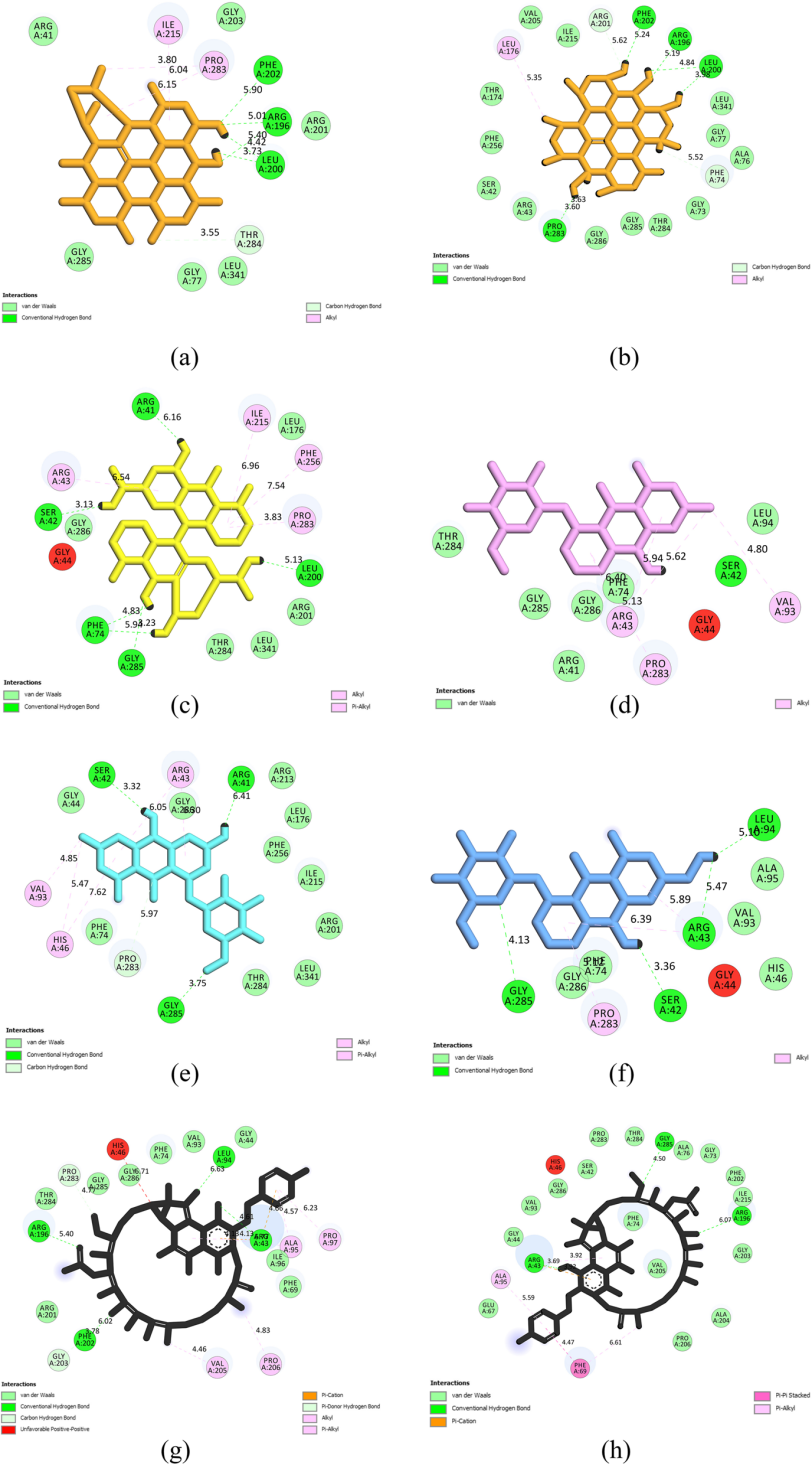
Two-dimensional view of the top-ranked anthraquinones and the reference drug in this study inside the RIFMO active site. **a** Hypericin before MD simulation, **b** hypericin after MD simulation, **c** sennidin B, **d** pulmatin, **e** emodin 8-glucoside, **f** aloe-emodin 8-glucoside, **g** RIF before MD simulation, and **h** RIF after MD simulation. RIFMO, rifampicin monooxygenase; RIF, rifampicin

The DSV tool facilitated exploring interactions between RIF, the most potent RIFMO inhibitors, and residues within the enzyme’s catalytic cleft. Before MD simulation, among the investigated anthraquinones, hypericin exhibited the highest frequency of forming hydrogen bonds (*n* = 3), while emodin 8-glucoside established the most significant number of hydrophobic interactions (*n* = 5) with RIFMO residues. Notably, hypericin increased the number of hydrogen bonds to four after 100 ns of MD simulations. Throughout the MD simulation, stable hydrogen bonds were consistently observed between hypericin and Leu200. Before initiating MD simulations, RIF established four hydrogen bonds and seven hydrophobic interactions with the catalytic cleft of RIFMO. Following MD simulation, this drug exhibited a modification in its interactions, forming two hydrogen bonds and five hydrophobic interactions with the receptor. The interactions between the highest-ranked anthraquinones, RIF, and the active site of RIFMO are detailed in Table 4, with the exclusion of hydrogen bonds exceeding a distance of 5 Å. Figure 4 provides a two-dimensional representation of the interactions between the top-ranked anthraquinones, RIF, and the active site of RIFMO.

**Table 4:** The interaction modes between the RIFMO catalytic cleft, the highest-ranked anthraquinones, and RIF as a positive control inhibitor

Ligand name	Hydrogen bond (distance Å)	Hydrophobic interaction (distance Å)	Electrostatic (distance Å)
Hypericin (before MD)	Leu200 (3.73, 4.42), Thr284 (3.55)	Pro283 (3.80, 6.16), Ile215 (6.04)	NA
Hypericin (after MD)	Leu200 (3.98, 4.84), Pro283 (3.6, 3.63)	Leu176 (5.35)	NA
Sennidin B	Ser42 (3.13), Phe74 (4.83)	Arg43 (6.54), Ile215 (6.96), Phe256 (7.54), Pro283 (3.83)	NA
Pulmatin	Ser42 (3.43)	Pro283 (5.13), Arg43 (5.94, 5.62), Val93 (4.80)	NA
Emodin 8-glucoside	Ser42 (3.32), Gly285 (3.75)	Val93 (4.85), His46 (5.47, 7.62), Arg43 (6.05, 6.30)	NA
Aloe-emodin 8-glucoside	Ser42 (3.36), Gly285 (4.13)	Arg43 (5.89, 6.39), Pro283 (5.12)	NA
Rifampicin (before MD)	Pro283 (4.77), Arg43 (4.13, 4.61), Gly203 (3.78)	Val205 (4.46), Pro206 (4.83), Pro97 (6.23), Ala95 (4.57, 6.77), Arg43 (4.66, 4.13)	Arg43 (4.13, 4.66)
Rifampicin (after MD)	Arg43 (3.69), Gly285 (4.5)	Arg43 (3.92, 3.92), Phe69 (4.47, 6.61), Ala95 (5.59)	Arg43 (3.92)

*RIFMO* rifampicin monooxygenase, *RIF* rifampicin, *NA* not available

### Drug likeness and ADMET

All compounds under examination, including anthraquinones and RIF, were subjected to drug-likeness analysis based on Lipinski et al.’s [47] study outlining the RO5. Data analysis showed that hypericin, sennidin A, and sennidin B displayed numerous violations of Lipinski’s RO5, indicating their unsuitability for oral drug administration. Remarkably, the standard drug RIF also exhibited three breaches of the RO5 criteria, mirroring the observations made with hypericin. This insight suggests that despite its extensive clinical usage, RIF does not adhere to Lipinski’s RO5 guidelines for optimal oral bioavailability (Table 5).

**Table 5:** Physicochemical properties of ligands concerning Lipinski’s rule of five

Ligand name	Molecular weight (g/mol)	LogP	Hydrogen bond donor count	Hydrogen bond acceptor count	Number of violations from RO5	Orally active drug
Hypericin	504.4	5.7	6	8	3	No
Sennidin B	538.5	4.8	6	10	2	No
Pulmatin (chrysophanol-8–*0*-glucoside)	416.4	1.3	5	9	0	Yes
Emodin 8-glucoside	432.4	0.9	6	10	1	Yes
Aloe-emodin 8-glucoside	432.4	0	6	10	1	Yes
Rhein	284.22	2.2	3	6	0	Yes
Physcion	284.26	3	2	5	0	Yes
Sennidin A	538.5	4.8	6	10	2	No
Emodic acid	300.22	1.9	4	7	0	Yes
Alizarin	240.21	3.2	2	4	0	Yes
Aloe-emodin	270.24	1.8	3	5	0	Yes
Chrysophanol	254.24	3.5	2	4	0	Yes
Emodin	270.24	2.7	3	5	0	Yes
Purpurin	265.21	2.9	3	5	0	Yes
Rhodoptilometrin	314.25	2.2	4	7	0	Yes
Danthron	240.21	3.2	2	4	0	Yes
Obtusifolin	284.26	3	2	5	0	Yes
Knipholone	434.4	4.2	4	8	0	Yes
Rubiadin	254.24	3.1	2	4	0	Yes
Nordamnacanthal	268.22	2.7	2	5	0	Yes
Damnacanthal	282.25	2.5	1	5	0	Yes
Rifampicin (Ctrl +)	822.9	4.9	6	15	3	No

*LogP* the logarithm of the partition coefficient between n-octanol and water, *RO5* rule of five

The comprehensive analysis in Table 6 indicates a robust safety profile for all anthraquinone compounds examined, including the standard drug RIF, regarding carcinogenicity prediction. However, notable distinctions emerge among these compounds when evaluating ADME (absorption, distribution, metabolism, and excretion). Hypericin, sennidin A, sennidin B, pulmatin, emodin 8-glucoside, aloe-emodin 8-glucoside, knipholone, and RIF demonstrate limited potential for gastrointestinal absorption. Furthermore, alizarin, chrysophanol, danthron, and rubiadin exhibit successful blood–brain barrier penetration. Predictive analyses suggest pulmatin, emodin 8-glucoside, and RIF could induce drug resistance via g-protein-coupled receptor inhibition. Additionally, emodin 8-glucoside, aloe-emodin 8-glucoside, rhein, emodic acid, and RIF are forecasted to lack inhibitory effects on cytochrome P450 enzymes, indicating favorable metabolic pathways within the human system.

**Table 6:** Predicted ADMET profiles for all investigated ligands in the study

Ligand name	GI abs	BBB permeant	P-gp substrate	CYP1A2 inhibitor	CYP2C19 inhibitor	CYP2C9 inhibitor	CYP2D6 inhibitor	CYP3A4 inhibitor	Carcinogenesis
Hypericin	Low	No	No	No	Yes	Yes	No	No	High safety
Sennidin B	Low	No	No	No	No	Yes	No	No	High safety
Pulmatin (chrysophanol-8–*0*-glucoside)	Low	No	Yes	No	No	Yes	No	No	High safety
Emodin 8-glucoside	Low	No	Yes	No	No	No	No	No	High safety
Aloe-emodin 8-glucoside	Low	No	No	No	No	No	No	No	High safety
Rhein	High	No	No	No	No	No	No	No	High safety
Physcion	High	No	No	Yes	No	Yes	No	Yes	High safety
Sennidin A	Low	No	No	No	No	Yes	No	No	High safety
Emodic acid	High	No	No	No	No	No	No	No	High safety
Alizarin	High	Yes	No	Yes	No	No	No	Yes	High safety
Aloe-emodin	High	No	No	Yes	No	No	No	Yes	High safety
Chrysophanol	High	Yes	No	Yes	No	No	No	Yes	High safety
Emodin	High	No	No	Yes	No	No	No	Yes	High safety
Purpurin	High	No	No	Yes	No	No	No	Yes	High safety
Rhodoptilometrin	High	No	No	No	No	No	No	Yes	High safety
Danthron	High	Yes	No	Yes	No	No	No	Yes	High safety
Obtusifolin	High	No	No	Yes	No	Yes	No	Yes	High safety
Knipholone	Low	No	No	No	No	Yes	No	Yes	High safety
Rubiadin	High	Yes	No	Yes	No	No	No	Yes	High safety
Nordamnacanthal	High	No	No	No	No	No	No	Yes	High safety
Damnacanthal	High	No	No	Yes	No	No	No	Yes	High safety
Rifampicin (Ctrl +)	Low	No	Yes	No	No	No	No	No	High safety

*GI* gastrointestinal, *abs* absorption, *BBB* blood–brain barrier, *P-gp* p-glycoprotein, *CYP* cytochrome p-450

## Discussion

Emerging evidence supports the role of RIFMO in antibiotic resistance by inactivating rifampicin through oxidative reactions. Structural studies have provided valuable insights into the mechanism of RIFMO-mediated resistance. Targeting RIFMO could be a potential strategy to combat antibiotic resistance [7, 8]. Anthraquinones, organic compounds, have exhibited promise in fighting *tuberculosis* [14, 15]. This study employed in silico approaches to assess the potential inhibitory impacts of selected anthraquinones on the RIFMO catalytic domain. Subsequently, a comparative analysis was conducted between these outcomes and those of RIF, employed as a reference compound.

The study’s findings reveal that five of the analyzed anthraquinones exhibited notably favorable Δ*G*_binding_ values concerning RIFMO, all falling below the − 10 kcal/mol threshold. This underscores their considerable effectiveness as RIFMO inhibitors within the examined compounds’ scope. Notably, the calculated Δ*G*_binding_ value of − 9.92 kcal/mol between RIFMO and the reference inhibitor serves as a benchmark, and the anthraquinones featured in this investigation surpassed this benchmark, indicating their favorable binding free energy to the RIFMO active site.

Of particular significance is the identification of hypericin as the most potent RIFMO inhibitor, which displayed a remarkable Δ*G*_binding_ value of − 12.41 kcal/mol and a deficient *K*i value in the picomolar range. Sennidin B emerged as the study’s second most potent RIFMO inhibitor, with a binding energy of − 10.79 kcal/mol and a *K*i value of 12.31 nM. The computed binding energy between pulmatin (chrysophanol-8–0-glucoside) and the RIFMO active site was − 10.65 kcal/mol, while the binding energies between emodin 8-glucoside and aloe-emodin 8-glucoside with RIFMO were − 10.61 and − 10.6 kcal/mol, respectively. These results underscore the notable inhibitory potential of these anthraquinones and their efficacy in targeting the RIFMO enzyme. Table 7 summarizes key findings for the top-ranked anthraquinones.

**Table 7:** A summary table of the key findings for the top-ranked anthraquinones

Compound	Δ*G*_binding_ (kcal/mol)	*K*i value	Key interactions with RIFMO	Orally active drug
Hypericin	− 12.41	798.99 pM	H-bonds: Leu200, Thr284 Hydrophobic: Pro283, Ile215	No
Sennidin B	− 10.79	12.31 nM	H-bonds: Ser42, Phe74 Hydrophobic: Arg43, Ile215, Phe256, Pro283	No
Pulmatin	− 10.65	15.54 nM	H-bond: Ser42 Hydrophobic: Pro283, Arg43, Val93	Yes
Emodin 8-glucoside	− 10.61	16.69 nM	H-bonds: Ser42, Gly285 Hydrophobic: Val93, His4, Arg43	Yes
Aloe-emodin 8-glucoside	− 10.6	16.95 nM	H-bonds: Ser42, Gly285 Hydrophobic: Arg43, Pro283	Yes
RIF	− 9.92	53.32 nM	H-bonds: Pro283, Arg43, Gly203 Hydrophobic: Val205, Pro206, Pro97, Ala95, Arg43 Electrostatic: Arg43	No

*RIFMO* rifampicin monooxygenase, *RIF* rifampicin, *K*i inhibition constant

Hypericin, a naturally occurring anthraquinone, has garnered significant interest due to its multifaceted pharmacological properties, encompassing anticancer, antibacterial, antiviral, and neuroprotective effects [50]. There is growing evidence to suggest the antibacterial efficacy of hypericin against *Staphylococcus aureus* (*S. aureus*). Whether in water-soluble formulations or as part of plant extracts, hypericin has consistently demonstrated the ability to significantly reduce bacterial counts and inhibit the growth of *S. aureus*. In their study, Engelhardt et al. [51] assessed the effectiveness of water-soluble formulations of hypericin in eradicating *S. aureus* through antibacterial photodynamic therapy. The authors observed that incubating *S. aureus* with 100-nM hypericin for 5 min, followed by 30 min of illumination, substantially reduced bacterial counts. Likewise, a concentration of 300-nM hypericin, when incubated for 5 min and followed by 30 min of illumination, exhibited superior efficacy compared to a 10-min illumination at a higher light intensity. These findings suggest that hypericin holds promise as a potential treatment for *S. aureus* infections.

The comparative analysis conducted on hypericin, concerning rifampicin, a primary drug in *tuberculosis* treatment, highlights the superior binding affinity of hypericin to the RIFMO active site. Despite structural disparities between rifampicin and hypericin, the notably enhanced binding affinity displayed by hypericin suggests its promise as a lead compound warranting further investigation in *tuberculosis* drug development. In a related investigation, Chopra et al. [52] conducted a research study focusing on the development and potential application of a novel imaging agent, specifically 64Cu-labeled bis-1,4,7,10-tetraazacyclododecane-N,N′,N,N′-tetraacetic acid (DOTA)-conjugated hypericin, within the context of photothermal ablation (PTA) therapy for cancer treatment. Their experimental findings provided evidence supporting the utility of this conjugated hypericin in evaluating the response to PTA therapy in a preclinical cancer model. The authors concluded that hypericin, primarily when conjugated with specific agents like DOTA for imaging purposes, presents a promising avenue for enhancing the assessment of cancer therapy efficacy. However, while Chopra et al. [52] did not directly address the query regarding the anti-*tuberculosis* effects of hypericin, their research indirectly sheds light on a potential area of interest for hypericin’s application in distinguishing between cancerous lesions and *tuberculosis* infections. This suggests the necessity for further exploration into hypericin’s potential utility in this context.

Additionally, Bahmani et al. [53] explored the antibacterial properties of hydroalcoholic extracts of *Hypericum perforatum*, which contains hypericin, against *S. aureus*. Their research demonstrated that hypericin was one of the predominant compounds within the extract. The minimum inhibitory concentration (MIC) and minimum bactericidal concentration (MBC) of hypericin against *S. aureus* were determined to be 312.5 µg/mL and 2500 µg/mL, respectively, underscoring the antibacterial activity of hypericin against *S. aureus*. Furthermore, Khaksarian et al. [54] investigated the antibacterial properties of titanium dioxide nanoparticles synthesized using *Hypericum perforatum* extract and hypericin against *S. aureus*. The authors established that the MIC of hypericin was 250 µg/mL, further substantiating the antibacterial potential of hypericin against *S. aureus*. Before the commencement of the MD simulation, hypericin established three hydrogen bonds and three hydrophobic interactions with critical residues, namely Leu200, Ile215, Pro283, and Thr284, situated within the active site of the RIFMO. After the MD simulation, this anthraquinone compound demonstrated an enhancement, with the formation of four hydrogen bonds and one hydrophobic interaction involving residues denoted as Leu176, Leu200, and Pro283, located within the catalytic domain of the RIFMO.

Chrysophanol, a natural anthraquinone compound, has dramatically benefited food and pharmaceuticals. As highlighted in a comprehensive review by Xie et al. [55], chrysophanol boasts a wide-ranging spectrum of pharmacological attributes, encompassing anticancer, antioxidant, neuroprotective, antibacterial, antiviral, and lipid-regulating properties. These multifaceted effects suggest a potential therapeutic role for chrysophanol in addressing a variety of ailments. In the context of its antibacterial capabilities, chrysophanol has exhibited inhibitory prowess against a diverse array of bacterial strains. Studies have elucidated its efficacy against gram-positive and gram-negative bacteria, including notorious pathogens such as *S. aureus*, *Escherichia coli* (*E. coli*), and *Pseudomonas aeruginosa* (*P. aeruginosa*) [55]. The precise mechanism underlying chrysophanol’s antibacterial activity remains an area of ongoing exploration. However, it is posited that chrysophanol may disrupt the structural integrity of bacterial cell membranes, thereby inducing cell lysis and eventual demise. Additionally, chrysophanol may impede bacterial growth by interfering with essential enzymes or proteins, further contributing to its antibacterial effects. Chrysophanol-8-O-glucoside displayed the establishment of a single hydrogen bond and the participation in four hydrophobic interactions, engaging with specific amino acid residues, namely Ser42, Arg43, Val93, and Pro283, all strategically positioned within the active site of the RIFMO enzyme.

In a study by Duan et al. [56], the antibacterial potential of naturally chlorinated emodin, specifically 1,3,8-trihydroxy-4-chloro-6-methyl-anthraquinone (CE), was explored against gram-positive bacteria, including drug-resistant strains such as methicillin-resistant *S. aureus* (MRSA) and vancomycin-resistant *Enterococcus faecium* (VRE). The investigation revealed that CE effectively impeded the growth of gram-positive bacteria, particularly demonstrating efficacy against drug-resistant strains. The underlying mechanism of CE’s action involved damaging bacterial cell membranes and inducing DNA condensation. These findings underscored the promise of CE as a natural antibacterial agent against drug-resistant bacteria. In a separate study conducted by Otieno et al. [57], the effects of aloe-emodin (AE) in combination with photodynamic therapy (PDT) on toxins associated with sepsis produced by gram-positive bacteria were examined. The research focused on the impact of AE and PDT on bacterial strains such as *Enterococcus faecalis*, *S. aureus*, and *Streptococcus pneumoniae*. The results demonstrated that AE-mediated PDT effectively reduced bacterial survival, inhibited colony and biofilm formation, and mitigated the production of pro-inflammatory cytokines, hemolytic activities, and the expression of toxins associated with sepsis. Otieno et al. [57] thus highlighted the potential of AE and PDT in attenuating sepsis-associated toxins in gram-positive bacteria.

Moreover, in a study by Nguyen et al. [58], the role of chitosan in enhancing the solubility and antibacterial activity of emodin against drug-resistant bacterial strains was investigated. The researchers formulated a combination of chitosan and emodin (EMD/CS) to improve the solubility of emodin. The EMD/CS formulation exhibited increased solubility of emodin and displayed synergistic antibacterial effects against drug-resistant bacterial strains, including MRSA and *Escherichia coli* O157:H7 (*E. coli* O157:H7). Nguyen et al. [58] demonstrated the potential of the EMD/CS formulation as a candidate for treating infectious diseases caused by drug-resistant bacterial pathogens. Emodin 8-glucoside and aloe-emodin 8-glucoside each established dual hydrogen bonds with Ser42 and Gly285, strategically located within the active site of the RIFMO enzyme. In addition to these hydrogen bonds, emodin 8-glucoside displayed five distinct hydrophobic interactions, engaging Arg43, His46, and Val93. In comparison, aloe-emodin 8-glucoside demonstrated three hydrophobic interactions, specifically involving Arg43 and Pro282, all within the confines of the RIFMO active site.

Despite the structural distinctions (rifampicin is a chiral, multi-heterocyclic drug, while hypericin is a multi-carbocyclic compound), the notably superior binding affinity exhibited by hypericin in contrast to RIF implies its promising potential as a lead compound warranting further exploration. The detailed analysis of RIFMO-inhibitor interactions provides valuable insights into the structural basis of the inhibition mechanism, informing the design of more potent and selective inhibitors targeting the RIFMO enzyme to develop effective antimicrobial therapies. Furthermore, the MD simulations yielded valuable insights into the interactions between the RIFMO enzyme and two small molecule inhibitors, hypericin and RIF. Before the MD simulations, RIF established several vital interactions within the RIFMO catalytic cleft, including hydrogen bonds with Pro283, Arg43, and Gly203 and hydrophobic interactions with Val205, Pro206, Pro97, Ala95, and Arg43. An electrostatic interaction with the Arg43 residue was also observed for RIF before and after the MD simulations.

In contrast, hypericin exhibited a different binding mode with RIFMO before the MD simulations, forming hydrogen bonds with Leu200 and Thr284 and engaging in hydrophobic interactions with Pro283 and Ile215. No electrostatic interactions were detected for the hypericin-RIFMO complex. Post-MD simulations, the binding interactions for both inhibitors changed. For RIF, hydrogen bonding interactions formed with Arg43 and Gly285, while the hydrophobic interaction pattern shifted to involve Arg43, Phe69, and Ala95, with the persistent electrostatic interaction with Arg43 still observed. In the hypericin-RIFMO complex, hydrogen bonding interactions with Leu200 and Pro283 were demonstrated after the MD simulations, with hydrophobic interactions primarily involving Leu176, indicating a shift in the binding mode compared to the pre-MD state.

These findings suggest that RIF and hypericin can bind to the RIFMO enzyme but through distinct interaction patterns. RIF forms a stable set of interactions, including hydrogen bonding, hydrophobic interactions, and electrostatic interactions, contributing to its potency as an inhibitor. In contrast, hypericin’s binding interactions, including hydrogen bonding, rely more on hydrophobic contacts. Moreover, the hydrogen bond connections between hypericin at Leu200 and RIF at Arg43 remained unchanged throughout the MD simulation. The hydrophobic interactions between RIF at Arg43 and RIF at Ala95 and the electrostatic interaction between RIF and Arg43 showed stability over a 100-ns computer simulation.

Based on the interactions observed between the top-ranked anthraquinones and the residues of RIFMO, proposed mechanisms rooted in structure–activity relationships (SARs) elucidate how anthraquinones function as inhibitors of RIFMO:

- a) Hypericin
  Hypericin engages in hydrogen bonding interactions facilitated by its hydroxyl groups with specific residues of RIFMO, while its aromatic rings contribute to hydrophobic interactions. The presence of multiple hydroxyl groups and aromatic rings in hypericin affords robust binding to the RIFMO active site, resulting in substantial inhibitory activity. Among the tested anthraquinones, hypericin demonstrated the most negative binding free energy to RIFMO, underscoring its potential as a potent inhibitor. The synergistic interplay between hydrogen bonding and hydrophobic interactions augments its inhibitory efficacy.
- b) Sennidin B
  Sennidin B similarly forms hydrogen bonds through its hydroxyl groups, with additional contributions from its carbonyl group, while its aromatic rings participate in hydrophobic interactions. The arrangement of hydroxyl and carbonyl groups in sennidin B facilitates moderate binding to RIFMO, albeit with lower affinity compared to hypericin.
- c) Pulmatin
  Pulmatin primarily engages in hydrophobic interactions via its aromatic rings and methyl group. Aromatic rings and a methyl group in pulmatin contribute to its moderate binding free energy to RIFMO. Pulmatin exhibits higher binding free energy relative to hypericin and sennidin B, indicating a weaker inhibitory effect.
- d) Emodin 8-glucoside and *aloe*-emodin 8-glucoside
  Both compounds interact with RIFMO through hydrogen bonding facilitated by hydroxyl and carbonyl groups, while their aromatic rings contribute to hydrophobic interactions. Glucose moieties in emodin 8-glucoside and aloe-emodin 8-glucoside influence their binding free energy, with hydroxyl and carbonyl groups playing pivotal roles. These compounds demonstrate higher binding free energy than hypericin, sennidin B, and pulmatin, signifying weaker inhibitory potential.

One limitation of the current study is the lack of a structurally similar reference compound to the screened anthraquinone derivatives. The availability of a RIFMO inhibitor with a closer structural resemblance to the anthraquinones would facilitate the development of a SAR. Despite this limitation, the comparative analysis with RIF still provides valuable insights into the potential of the anthraquinone compounds as RIFMO inhibitors.

Building upon the promising computational results of this study, several crucial next steps are warranted to validate and develop these findings further. Firstly, experimental validation through in vitro assays is essential to confirm the inhibitory effects of the identified anthraquinones, particularly hypericin, against RIFMO. This could involve enzyme inhibition assays and MIC tests against rifampicin-resistant bacterial strains. Secondly, SAR studies should be conducted to optimize the lead compounds, potentially enhancing their potency and pharmacokinetic properties. This might involve synthesizing and testing structural analogs of hypericin and other top-performing anthraquinones. Thirdly, in vivo studies in appropriate animal models would be crucial to assess these compounds’ efficacy and safety profiles. Finally, if preclinical studies yield positive results, clinical trials could be initiated to evaluate the potential of these RIFMO inhibitors as adjuvants in combination therapy with existing antibiotics. These steps would pave the way for potential clinical applications in combating antibiotic resistance, particularly in treating tuberculosis and other bacterial infections where rifampicin resistance is a concern.

## Conclusion

This study highlights substantial binding affinities of hypericin, sennidin B, pulmatin, emodin 8-glucoside, and aloe-emodin 8-glucoside to the RIFMO active site. Especially noteworthy is hypericin, which exhibits exceptional potency, reflected in a Δ*G*_binding_ score of − 12.11 kcal/mol, as well as a Ki value in the picomolar range (798.99 pM). Furthermore, it demonstrates remarkable stability throughout a 100-ns MD simulation, evidenced by an RMSD of 0.55 nm. These findings offer valuable insights for researchers exploring novel drug therapies, especially for infections like *tuberculosis*. While the new legislation has removed the mandatory requirement for animal testing, it remains crucial to validate the inhibitory effects of these compounds through rigorous in vitro experimentation, followed by subsequent clinical trials. This stepwise approach is essential to ensure the safety and efficacy of new pharmaceutical candidates before they can be approved for human use.

## Supplementary Information

Supplementary Material 1: Supplementary File 1. Details of docking result files for top-ranked anthraquinones and the reference drug.

## Data Availability

Data is provided within the manuscript and supplementary information file.
